# Single-Cell STAT5 Signal Transduction Profiling in Normal and Leukemic Stem and Progenitor Cell Populations Reveals Highly Distinct Cytokine Responses

**DOI:** 10.1371/journal.pone.0007989

**Published:** 2009-11-24

**Authors:** Lina Han, Albertus T. J. Wierenga, Marjan Rozenveld-Geugien, Kim van de Lande, Edo Vellenga, Jan Jacob Schuringa

**Affiliations:** 1 Department of Hematology, University of Groningen, University Medical Center Groningen, Groningen, The Netherlands; 2 Department of Hematology, The First Clinical College of Harbin Medical University, Harbin, China; LMU University of Munich, Germany

## Abstract

**Background:**

Signal Transducer and Activator of Transcription 5 (STAT5) plays critical roles in normal and leukemic hematopoiesis. However, the manner in which STAT5 responds to early-acting and lineage-restricted cytokines, particularly in leukemic stem/progenitor cells, is largely unknown.

**Methodology/Principal Findings:**

We optimized a multiparametric flow cytometry protocol to analyze STAT5 phosphorylation upon cytokine stimulation in stem and progenitor cell compartments at a single-cell level. In normal cord blood (CB) cells, STAT5 phosphorylation was efficiently induced by TPO, IL-3 and GM-CSF within CD34^+^CD38^−^ hematopoietic stem cells (HSCs). EPO- and SCF-induced STAT5 phosphorylation was largely restricted to the megakaryocyte-erythroid progenitor (MEP) compartment, while G-CSF as well IL-3 and GM-CSF were most efficient in inducing STAT5 phosphorylation in the myeloid progenitor compartments. Strikingly, mobilized adult peripheral blood (PB) CD34^+^ cells responded much less efficiently to cytokine-induced STAT5 activation, with the exception of TPO. In leukemic stem and progenitor cells, highly distinct cytokine responses were observed, differing significantly from their normal counterparts. These responses could not be predicted by the expression level of cytokine receptors. Also, heterogeneity existed in cytokine requirements for long-term expansion of AML CD34^+^ cells on stroma.

**Conclusions/Significance:**

In conclusion, our optimized multiparametric flow cytometry protocols allow the analysis of signal transduction at the single cell level in normal and leukemic stem and progenitor cells. Our study demonstrates highly distinctive cytokine responses in STAT5 phosphorylation in both normal and leukemic stem/progenitor cells.

## Introduction

Hematopoiesis is largely regulated by signaling cascades that are activated by a wide variety of cytokines [1]. The signals that emanate from cytokine receptors are translated into specific cellular responses via activation of transcription factors that induce expression of unique sets of target genes. One family of such transcription factors is the Signal Transducer and Activator of Transcription (STAT) family, which consists of 7 members, STAT1-6 whereby STAT5A and STAT5B are encoded by two separate genes. STAT5 is widely expressed throughout the hematopoietic system, targeting genes that have been associated with proliferation, anti-apoptosis or differentiation [2]–[4]. Loss-of-function studies demonstrated that long-term repopulating activity of hematopoietic stem cells (HSC) was impaired in STAT5A-deficient HSCs [5]–[7]. During steady-state hematopoiesis, conditional deletion of STAT5 in nonablated adult mouse gradually reduced the HSC pool size and caused loss of HSC quiescence [8]. Our previous studies on STAT5 downregulation also showed impaired maintenance and expansion of primitive human hematopoietic stem and progenitor cells [9], [10]. Stress-induced erythropoiesis was severely impaired in STAT5^−/−^ mice [11], and appropriated STAT5 signaling was also required for maintaining a normal lymphoid-myeloid balance [12]. Conversely, in gain-of-function studies overexpression of activated STAT5A in CB CD34^+^ cells resulted in enhanced stem cell self-renewal and erythroid commitment, at the expense of normal myelopoiesis and megakaryocyte development [13]–[15]. Introduction of a persistently activated STAT5A mutant (S711F) enabled erythropoiesis in an EPO-independent manner [16]. Together these studies demonstrated critical roles for STAT5 in various hematopoietic compartments.

Constitutive STAT5 signaling has been identified in the pathogenesis of various hematological malignancies, including BCR-ABL-induced chronic myeloid leukemia (CML), acute myeloid leukemia (AML), acute lymphoid leukemia (ALL) and myeloproliferative disorders (MPDs) such as chronic myelomonocytic leukemia (CMML) and polycythemia vera (PV) [4]. In AML, constitutive STAT5 signaling is observed in the majority of cases, resulting from either mutations in upstream receptor tyrosine kinases such as FLT3 and c-KIT, or autocrine growth factor production [17]–[20]. In primary human AML CD34^+^ cells, lentiviral downregulation of STAT5 resulted in impaired long-term expansion and self-renewal on stroma [9].

Despite increasing evidence indicating a critical role for STAT5 in normal and leukemic hematopoiesis, little is known about how STAT5 responds to different early-acting and lineage-restricted cytokines. Since a lot of studies investigated STAT5 activity in bulk populations, it has been particularly unclear whether and when STAT5 is activated upon cytokine stimulation within individual cells in stem cell and progenitor compartments. Also, it has been unclear whether constitutive STAT5 activity is specifically present in leukemic stem cell-enriched populations, or predominantly within the non-self-renewing leukemic progeny. In the current study, we have optimized multiparametric FACS protocols in order to evaluate activation of the STAT5 signal transduction pathway in specific hematopoietic stem cell and progenitor subpopulations, both in normal human cord blood (CB) and peripheral blood (PB), as well as in primary AML patient samples. Our current study reveals highly distinct cytokine responses in normal and leukemic stem and progenitor subpopulations.

## Results

### Cytokine-Induced STAT5 Phosphorylation in Normal Stem/Progenitor Cells Derived from CB and PB Cells

To test the affinity and specificity of phospho-STAT5 (pSTAT5) antibodies for FACS procedures, the cytokine-dependent UT-7 cell line and BCR-ABL posititive K562 cell line were used. UT-7 cells were cytokine-depleted overnight followed by EPO stimulation over several time points. The kinetics of pSTAT5 observed by Western blotting (Fig. S1A) fitted well with that observed in FACS analysis (Fig. S1B and S1C). Constitutively activated STAT5 could be observed in the BCR-ABL-positive K562 cell line, whereby downregulation of pSTAT5 upon treatment with imatinib was observed in both Western blotting and FACS experiments (Fig. S1D-F). Furthermore, we wished to compare the staining properties of membrane markers necessary for discriminating stem cell and progenitor compartments in paraformaldehyde and methanol (F/M)-treated conditions versus non-fixed conditions. Antibodies against CD34, CD38, CD123, CD45RA and CD110 were titrated in CB CD34^+^ cells in both non-fixed and F/M-treated conditions to obtain optimal separation (all titration results are shown in Fig. S2). As previously described, these antibodies have previously been shown to discriminate between BFU-Us, CFU-GM and CFU-GEMM progenitor cells [21]–[23]. Various tested antibodies were insufficient in separating HSC, common myeloid progenitors (CMP), granulocyte-macrophage progenitors (GMP) and megakaryocyte-erythroid progenitors (MEP) under F/M-treated conditions, but ultimately we were able to identify a set of antibodies with which the various compartments could be visualized by FACS under F/M-treated conditions (Fig. S3).

**Figure 1:**
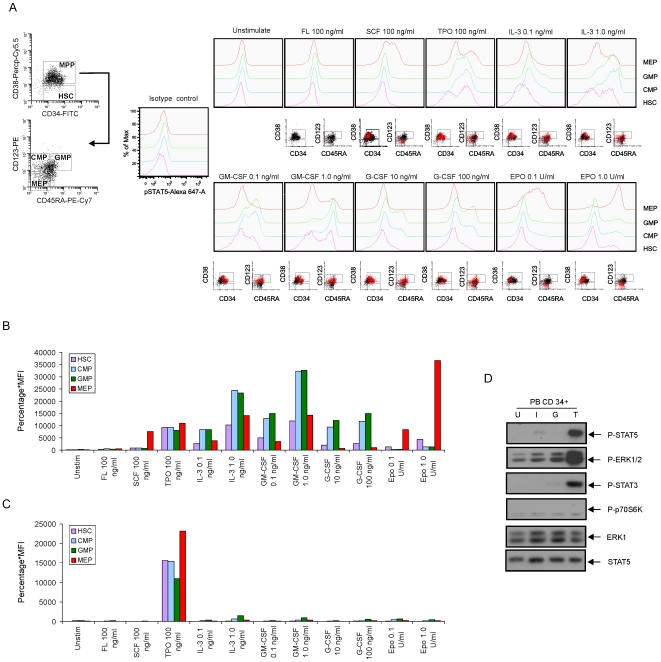
STAT5 phosphorylation in defined stem and progenitor cell populations in normal hematopoietic cells. (A) Normal CD34^+^ cells isolated from cord blood (CB) were expanded in HPGM supplemented with 100 ng/ml SCF, FL and TPO for 3 days. Cells were washed and cytokine-depleted in HPGM overnight and then stimulated with indicated cytokines for 15 minutes, followed by intracellular FACS. The scatter properties and gating schemes are shown. The red dots represent the cells with activated STAT5 upon stimulation. (B) Overview of cytokine responses in STAT5 activation in CB CD34^+^ cells quantified as the percentage of positive cells multiplied with the mean fluorescence intensity (MFI). (C) Normal CD34^+^ cells derived from mobilized peripheral blood (PB) were suspended in HPGM for 2 hours, followed by cytokine stimulation and intracellular FACS. (D) 1×10^6^/ml PB CD34^+^ cells were isolated and suspended in HPGM for 2 hours. Cells were stimulated with 1.0 ng/ml IL-3 (I), 100 ng/ml G-CSF (G) or 100 ng/ml TPO (T) for 15 minutes or left unstimulated (indicated as U), after which cell lysates were prepared and subjected to Western blotting using antibodies as indicated.

Our FACS protocol was first tested in CB CD34^+^ cells stimulated with FL, SCF, TPO, IL-3, GM-CSF, G-CSF and EPO for 15 minutes at different concentrations. As displayed in Figure 1A, EPO induced a strong STAT5 phosphorylation within the MEP compartment, while no STAT5 phosphorylation was observed within the GMP compartment. Conversely, G-CSF-induced STAT5 phosphorylation was easily detected within CMP and GMP compartments, but not within the MEP compartment. We quantified the STAT5 responses by multiplying the percentage of positive cells with the mean fluorescence intensity (% x MFI, displayed in Fig. 1B) whereby isotype controls were used to set the gates for negative populations. Thus, these data provide further validation for the gate definitions of the progenitor subpopulations in our FACS protocols. Furthermore, variable cytokine-induced STAT5 responses were observed within the different compartments. FL was not sufficient in activating STAT5 in either stem or progenitor cells, in line with previous observations [24]. SCF induced a strong activation of STAT5 in the MEP, but not in the HSC. Within the HSC compartments, STAT5 could only be efficiently activated by TPO and high concentrations of IL-3 and GM-CSF. IL-3 and GM-CSF also activated STAT5 in the CMP and GMP compartments, but much less efficiently in the MEP. TPO could activate STAT5 in the HSC, CMP and MEP, and to a lesser extent in the GMP (Fig. 1A and 1B).

To be able to compare cytokine responses in fetal versus adult stem/progenitor cells, we also studied STAT5 activation in G-CSF-mobilized PB CD34^+^ cells (Fig. 1C). Strikingly, PB CD34^+^ cells responded strongly to TPO stimulation in all cell compartments, comparable to CB, but none of the other cytokines was able to efficiently induce STAT5 phosphorylation at the tested concentrations in PB. The absence of STAT5 phosphorylation was also confirmed by Western blotting of the bulk CD34^+^ PB population (Fig. 1D). While TPO also induced strong phosphorylation of ERK1/2 and STAT3 in PB CD34^+^ cells, none of these pathways was activated by any of the other tested cytokines.

### Heterogeneity in Cytokine-Induced STAT5 Phosphorylation in AML Stem/Progenitor Cells

**Figure 2:**
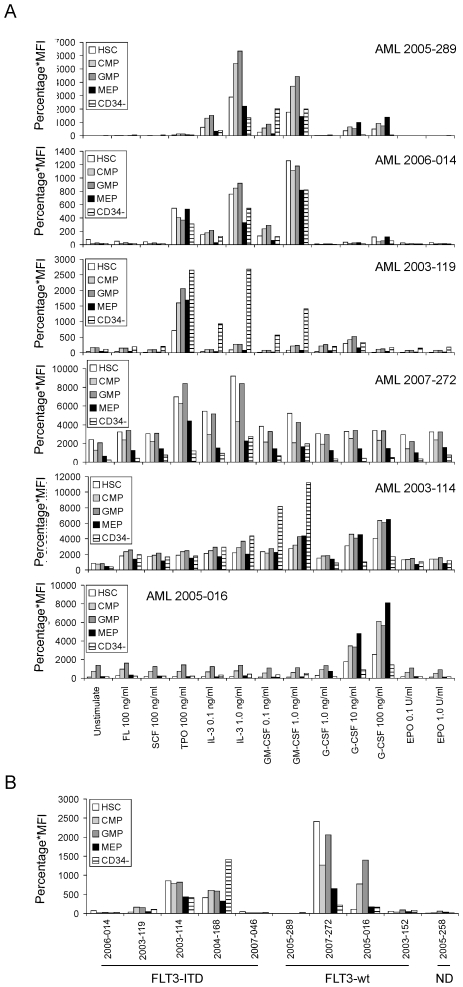
STAT5 phosphorylation in defined stem and progenitor cell populations in AML cases. AML PB mononuclear cells (MNCs) were thawed and suspended at 1.5×10^6^/ml in HPGM for 2 hours at 37°C. Cells were stimulated with cytokines for 15 minutes, followed by intracellular FACS. Panel (A) displays STAT5 phosphorylation in 6 AMLs with or without indicated cytokine stimulations. Panel (B) displays the basal levels of STAT5 phosphorylation (in unstimulated condition) of AML samples with FLT3-ITD versus FLT3-wild type. Data are presented as the multiplied value of the percentage of positive cells with the mean fluorescence intensity (MFI).

Next, we studied cytokine-induced STAT5 activation in primary AML samples (n = 10, Table 1). We followed the same definitions for HSC, CMP, GMP and MEP as in healthy CB and PB using CD34, CD38, CD123 and CD45RA as antigens, although it needs to be pointed out that these definitions of stem and progenitor compartments might not be valid in AML due to alterations in antigen expression. Nevertheless, for the sake of clarity we shall maintain this nomenclature throughout the manuscript. The CD34^−^ population within the mononuclear leukemic blast fraction was also included in our analyses, and the same cytokines were used to stimulate the AML PB cells as previously in healthy CB and PB. The results are shown in Figure 2A, and the raw FACS data were included as supplemental Figure S4A-J. In general, we observed that cytokine-induced STAT5 activation was highly variable throughout AML samples. When the complete CD34^+^ compartment was taken into account, 6 out of 10 AMLs responded to TPO, 6 out of 10 to G-CSF, 8 out of 10 to IL-3 and 4 out of 6 to GM-CSF (Table 1, Fig. 2A and Fig. S4A-J). No STAT5 phosphorylation was observed in response to SCF, FL or EPO in any of the investigated AML samples. When analyzing the HSC and MPP (CD34^+^CD38^+^) compartments, in many cases (6 out of 10) a strong shift from the MEP/CMP compartments towards the GMP was observed, possibly highlighting the myeloid character of the leukemias that were studied. Also since no EPO-induced STAT5 activation was observed in any of the investigated cases, the MEP fraction as defined by CD34^+^/CD38^+^/CD45RA^−^/CD123^−^ might not be a true representation of the erythroid progenitor compartment in AML. No explicit differences in cytokine-induced STAT5 responses were observed between the different HSC and MPP compartments, indicating that in case an AML responded to TPO, this was observed in both leukemic stem cells as well as in leukemic progenitors. Interestingly, in 3 out of 10 cases the most dominant cytokine-induced STAT5 phosphorylation was observed in the CD34^−^ fraction, particularly in response to IL-3 or GM-CSF (Table 1, Fig. 2A and Fig. S4A–J). Together, these data indicate that cytokine-induced STAT5 phosphorylation is distinct in AML cases, and differs compared to normal hematopoietic cells.

**Table 1:** Summary of clinical parameters of AML patients used in this study.

Patient ID	FAB	% CD34	Karyotype	FLT3-ITD	Responses to cytokines
2003-119	M0	80	N	+	c.a. TPO, IL-3, GM-CSF
2005-258	M0	73	-5q; +6	ND	G-CSF, IL-3
2006-014	M1	19	N	+	TPO, IL-3 GM-CSF
2005-016	M1	65	N	−	c.a., G-CSF
2004-168	M2	21	del (9) (q12;q22)	+	c.a., TPO, IL-3
2007-046	M2	20	N	+	TPO, G-CSF, IL-3
2003-152	M4	72	Inv (16)	−	TPO, G-CSF, IL-3
2005-289	M5	95	N	−	IL-3, GM-CSF, G-CSF
2003-114	M5	27	t(11;20)	+	c.a., G-CSF, GM-CSF
2007-272	M7	36	Complex	−	c.a. TPO, IL-3

AML cells derived from peripheral blood (PB); Percentage of CD34^+^ (% CD34) in the total AML mononuclear cell fraction; FAB, French-American-British classifications; FLT3-ITD (internal tandem duplication) was present (+) or absent (−) in the AML cells; ND, not determined; N, normal; c.a. constitutive activation of STAT5.

### The Basal Cytokine-Induced STAT5 Phosphorylation in AMLs with FLT3 Wild Type Versus FLT3-ITD

Constitutive STAT5 phosphorylation was observed in 5 AML cases (Fig. 2B, Table 1). Three of these AMLs contained FLT3 internal tandem duplication (FLT3-ITD) mutations while 2 had wild-type alleles (FLT3-wt). Interestingly, in 2 FLT3-ITD AMLs we could not observe constitutive activation of STAT5. In the FLT3-ITD AMLs, AML 2003-114 and 2004-168 showed constitutive STAT5 activity, which was observed in HSC and MPPs, as well as within the CD34^−^ fraction. In AML 2003-119 constitutive STAT5 activity was detected in the CMP/GMP, but not HSC compartment. In the FLT3-wt AMLs, the constitutive STAT5 activation varied between HSC and MPP fractions, whereby AML 2007-272 contained high constitutive STAT5 activation within the HSCs, while the STAT5 activation in AML 2005-016 was more pronounced within the CMP/GMP fractions. Remarkably, in all AMLs characterized by constitutive STAT5 activation, a strongly reduced cytokine response was observed (Fig. 2A and Fig. S4A-J), possibly suggesting the constitutive STAT5 signaling resulted in activation of negative feedback loops which render cells relatively insensitive to additional cytokine input. Indeed, in gene expression studies we observed that the negative feedback gene SOCS2 was specifically upregulated in these AMLs with constitutive STAT5 activity (data not shown).

### Cytokine Receptor Expression Does Not Predict Downstream Signaling in AML Stem/Progenitor Cells

**Figure 3:**
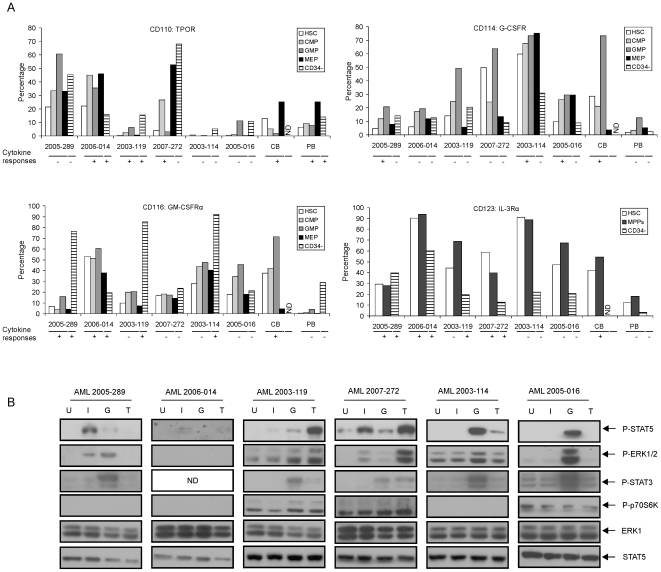
Cytokine and growth factor receptor expression in normal and leukemic cells. The expression of receptors for TPO (CD110), GM-CSF (CD116), IL-3 (CD123) and G-CSF (CD114) was analyzed by FACS. (A) MNCs from AML samples and CD34^+^ cells CB and PB were stained with antibodies against receptors, together with CD34, CD38, CD45RA and CD123 for 30 minutes. CD123 expression was analyzed in HSCs, MPPs (CD34^+^CD38^+^) and CD34^−^ populations. For CB cells, no CD34^−^ fraction was available. The cytokine responses for STAT5 activation in CD34^+^ versus CD34^−^ populations are also indicated (“+” indicates that STAT5 was activated by the respective cytokine; “−” indicates that STAT5 was not activated). (B) 1×10^6^/ml CD34^+^ cells were sorted by MoFlo from AML MNCs and suspended in HPGM for 2 hours. Cells were harvested after stimulation with 1.0 ng/ml IL-3 (I), 100 ng/ml G-CSF (G) or 100 ng/ml TPO (T) for 15 minutes or left unstimulated (indicated as U) for Western bloting. ND, not determined.

To investigate whether the differences in cytokine responses resulted from differences in expression levels of cytokine and growth factor receptors, we analyzed the surface marker expression by FACS (Fig. 3A). In normal cells, the TPOR (CD110) was mainly expressed in MEPs in both CB and PB CD34^+^ fraction, with some expression in HSCs, CMPs and GMPs as well. The TPO response was higher in MEP fraction and almost equal in HSCs, CMPs and GMPs in both PB and CB cells (Fig. 1A–1C). In CB CD34^+^ cells, the G-CSFR (CD114) was expressed in 73% of GMPs, 29% of HSC and 21% of CMPs, but not within MEPs. Similarly, the GM-CSFRα (CD116) was also predominantly expressed within the GMP compartment in CB CD34^+^ cells. The expression levels of G-CSFR and GM-CSFRα were much lower in PB CD34^+^ cells, which may explain the poor induction of STAT5 phosphorylation by these two cytokines in PB CD34^+^ cells (Fig. 3A, Fig. 1A–1C). IL-3Rα (CD123) was analyzed in HSC and MPPs (CD34^+^CD38^+^) because it was used to define CMPs/GMPs (as CD123^+^) and MEPs (as CD123^−^). In CB cells, CD123 was expressed in both HSCs and MPPs (42% and 54% respectively), which was in line with our observations regarding the IL-3-inducibility of STAT5 in these compartments (Fig. 1A). In PB CD34^+^ cells, CD123 was expressed at much lower levels (18% of MPPs). These results are consistent with a previous study, in which CD123 was identified as a marker for long-term repopulating cells in CD34^+^CD38^−^ fraction from bone marrow, cord blood and AMLs [25]. However, in other studies it was suggested that the CD34^+^/CD38^−^ HSC does not express CD123 [26]. We have observed that upon isolation, about 40% of CB CD34^+^/CD38^−^ cells express CD123 with low MFI, which was up by 3-fold upon culturing in HPGM supplemented with SCF, FL and TPO for 3–5 days (data not shown). To study the cytokine responses within the HSC compartment in more detail, we gated the CD34^+^/CD38^−^ cells into CD123^+^ and CD123^−^ compartments and analyzed cytokine-induced STAT5 phosphorylation. As shown in Supplemental Figure S5, the CD34^+^/CD38^−^/CD123^−^ compartment was unresponsive to IL-3, GM-CSF or G-CSF, while STAT5 was readily activated by these cytokines within the CD34^+^/CD38^−^/CD123^+^ compartment. Only TPO and EPO were able to activate STAT5 within CD34^+^/CD38^−^/CD123^−^ cells.

We also tested cytokine receptor expression in 6 AML samples. In general, the receptor expression level did not predict the level of cytokine-induced STAT5 activation (Fig. 3A, cytokine responses are indicated below the graphs). For example, AML 2003-119 was highly responsive to TPO stimulation, but expressed only relatively low levels of the TPOR. AML 2005-016 was highly G-CSF-responsive, but its G-CSFR expression was similar or even lower compared to e.g. AML 2003-119 and AML 2007-272, which didn't respond to G-CSF. Strikingly, a high level of GM-CSFRα expression was observed in CD34^−^ cells in the majority of AML cases, which did correlate with a strong GM-CSF-induced STAT5 phosphorylation within those cells (Fig. 2A and 3A). Since some cytokines were unable to induce STAT5 phosphorylation in some cases, we tested whether additional pathways could be activated in those AMLs in order to verify whether the receptors would be functional. Activation of the RAS/MEK/ERK, mTOR//4E-BP1/p70S6k and STAT3 signal transduction pathways were analyzed by Western blotting in the bulk CD34^+^ compartment given their importance in AML cell survival [27], [28]. We observed that all cytokine-induced STAT5 phosphorylation patterns that we observed by intracellular FACS were recapitulated in our Western blot analyses (Fig. 3B). While the constitutve STAT5 phosphorylation in AML 2007-272 was readily detected by Western blotting, the basal levels of pSTAT5 of AML 2003-119, 2003-114 and 2005-016 were not detected although constitutive levels were found in FACS analysis, reflecting a higher sensitivity of FACS compared Western blotting. Furthermore, we observed that in cases where e.g. G-CSF was unable to activate STAT5, STAT3 or ERK phosphorylation was readily induced (AML 2005-289 and AML 2003-119), indicating that in those cases the receptor complexes were clearly able to induce downstream signal transduction.

### Differential Cytokine Requirements for Long-Term In Vitro Expansion of AML CD34^+^ Cells on Bone Marrow Stroma

**Figure 4:**
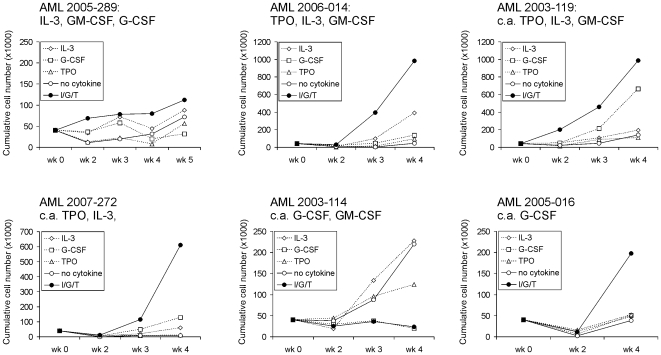
Variability in cytokine-dependent long-term expansion of AML CD34^+^ cells on MS5 stroma. 4×10^5^ AML CD34^+^ cells were sorted and plated in 12-well plates precoated with MS5 stromal cells. Cells were expanded in LTC medium supplemented with 20 ng/ml IL-3, G-CSF, TPO, combinations thereof, or without any cytokines. Cultures were demi-depopulated weekly for analysis. Weekly cumulative cell counts represented cells in suspension. The cytokines above the figures indicate which cytokines were able to induce STAT5 phosphorylation. c.a. indicates constitutive activation of STAT5.

Previously, we observed that STAT5 knockdown impaired long-term growth of leukemic CD34^+^ cells, indicating that STAT5 signaling was required for AML growth [9]. We now wondered whether AMLs would depend on specific cytokines for long-term proliferation on MS5 stromal cells, since heterogeneity was observed in AML cases with regard to their responses to cytokine-induced activation of STAT5. CD34^+^ cells were sorted from 6 AML samples and plated on MS5 stroma with IL-3, G-CSF, TPO and combinations thereof (Fig. 4). With the exception of AML 2005-289, long-term expanding cocultures could be established reaching 4- to 25-fold expansion, after 4 weeks of culture. All AMLs (except one: AML 2003-114) were dependent on cytokines for growth on stroma. Although AML 2003-119, AML 2007-272 and AML 2005-016 were characterized by constitutively activated STAT5, no long-term expansion was observed without cytokines. AML 2003-114, whereby long-term cultures could be established in the absence of cytokines, also contained constitutively activated STAT5. Interestingly, addition of G-CSF, either alone or in combinations with other cytokines, impaired long-term growth of this AML. Apparently, G-CSF activated certain signaling pathways that negatively regulated cell proliferation or induced differentiation. It is notable that AML 2003-119, in which STAT5 phosphorylation was strongly induced by TPO, but not G-CSF, no long-term cultures could be established with TPO alone. In contrast, addition of G-CSF was sufficient to initiate long-term expansion, although maximal expansion was reached in cultures where all cytokines were added. Together, these data indicate that heterogeneity exists in the cytokine requirement of AML CD34^+^ cells for long-term expansion in our in vitro stromal cocultures, and that in some cases constitutive STAT5 phosphorylation is not sufficient to confer cytokine-independent growth.

## Discussion

### STAT5 in Response to Cytokine in Normal HSC Versus MPPs

The current study demonstrates that intracellular FACS analysis is a very useful tool to dissect cytokine-induced signal transduction in single cells within normal and leukemic stem and progenitor compartments. Although various studies have shown that STAT5 can be activated by a plethora of cytokines, including FL, SCF, TPO, IL-3, G-CSF, GM-CSF and EPO, subsequently affecting the self-renewal and hematopoietic differentiation program, less has been revealed about how the STAT5 signaling pathway specifically responds to cytokine stimulation within the rare stem cells, or hematopoietic progenitor subfractions.

Loss-of-function studies have implicated STAT5 as an important regulator of HSC function [5]–[7], although little information has been available on the growth factors and cytokines that induce STAT5 within the human hematopoietic stem cell compartment. Within the human CB CD34^+^/CD38^−^ HSC compartment, STAT5 activation could be induced by TPO and high concentrations of IL-3 and GM-CSF. In murine HSCs, TPO-induced STAT5 signaling has been associated with enhanced self-renewal and improved competitive repopulation, in particular in Lnk^−/−^ HSCs which display hypersensitivity for TPO-induced STAT5 phosphorylation [29]. In human PBSCs, multilineage engraftment ability in NOD/SCID mice could be maintained by expansion in serum-free medium containing TPO, SCF and FL [30], and this cytokine cocktail is used in many studies to expand human CB CD34^+^ stem/progenitor cells e.g. in retroviral gene-targeting experiments [2], [22]. While we did observe that SCF was able to induce STAT5 activity in the MEP, no STAT5 phosphorylation was induced within the HSC. FL was unable to induce STAT5 phosphorylation in any of the hematopoietic compartments, in agreement with previously published observations [24], even though its receptor FLT3 was highly expressed at the HSC and CMP/GMP progenitor stages in human CD34^+^ cells from both bone marrow and cord blood [31]. Thus, it appears that in human stem cell expansion protocols it is particularly TPO that acts on HSCs, at least in terms of STAT5 signaling. In line with these observations, previous studies on STAT5-deficient bone marrow showed overlapping activities in HSC self-renewal capacity between STAT5AB^ΔN/ΔN^ and c-MPL^−^/^−^ mice, although not all the STAT5^−/−^ defects could be accounted for by loss of TPO signaling [6]. While mostly associated with signaling in myeloid progenitors, IL-3 and GM-CSF were able to activate STAT5 in HSCs as well. The role these factors fulfill in human HSCs has not been fully elucidated yet, but it has been observed that the responsiveness of STAT5AB^ΔN/ΔN^ HSCs to early-acting cytokines such as IL-3 was reduced, while the sensitivity to 5-fluorouracil was enhanced [5]. In agreement with previous published studies [25], [32], we find that the IL-3Rα (CD123) is indeed expressed within a subset of CD34^+^/CD38^−^ cells. When cytokine signaling was evaluated within the CD123^+^ and CD123^−^ HSC compartments we observed that the CD34^+^/CD38^−^/CD123^−^ compartment was unresponsive to IL-3, GM-CSF or G-CSF, while STAT5 was readily activated by these cytokines within the CD34^+^/CD38^−^/CD123^+^ compartment. Only TPO and EPO were able to activate STAT5 within CD34^+^/CD38^−^/CD123^−^ cells. Further studies will be required to determine whether both of these subpopulations contain in vivo repopulating activity.

In the erythroid progenitor compartment, STAT5 was activated by EPO, SCF and TPO. Gene knockout studies have revealed that STAT5 expression is required to prevent apoptosis during fetal development [33], surprisingly much more modest effects were observed on e.g. EPO and TPO signaling during adult steady-state hematopoiesis [34]. In the MEP, STAT5 fulfils an important anti-apoptotic role by upregulating Bcl-X_l_ [11], [35], [36], although a more direct role in initiating erythroid commitment might exist as well [15]. In myeloid progenitor cells, we find that STAT5 is activated by IL-3, GM-CSF and G-CSF. Since myelopoiesis appeared to be relatively unaffected in STAT5^−/−^ mice [12]. The exact role of STAT5 in myeloid progenitors remains elusive [12], although it is well possible that the signals initiated by e.g. IL-3 and GM-CSF regulate myeloproliferation or anti-apoptosis in these progenitor subsets as well [37]–[39].

Fetal stem/progenitor cells CD34^+^ cells isolated from cord blood might differ from adult stem/progenitor cells in various aspects, and therefore we included mobilized adult PB CD34^+^ cells in our analyses as well. Strikingly, only TPO was able to induce a robust STAT5 phosphorylation in PB CD34^+^ cells, while all other tested cytokines failed to activate STAT5 at the concentrations used in this study. Indeed, while the TPO receptor CD110 was expressed at comparable levels in CB and PB CD34^+^ cells, the expression of CD114, CD116 and CD123 was significantly lower in PB. However, it cannot be excluded that the in vivo applied G-CSF might have affected the receptor expression and down-stream signaling of the collected CD34^+^ cells. Exposure to recombinant human G-CSF causes down-regulation of G-CSF receptor and an increase in serum levels of growth factors including IL-8, vascular-endothelial growth factor (VEGF) and transforming growth factor beta (TGF-β), etc, subsequently modulating the cytokine responses and networks [40]. In MNCs isolated from BM, we did observe that STAT5 could be activated by G-CSF, TPO and IL-3 (data not shown). Our results are in line with a previous study in which primary CD34^+^CD41^+^ megakaryocytic progenitors isolated from mobilized PBSCs, whereby SCF stimulation alone did not induce detectable STAT5 activation, but prestimulation with SCF enhanced and prolonged TPO-induced STAT5 phosphorylation [41].

### STAT5 Signaling in Leukemic Stem and Progenitor Cells

In the vast majority of cases, leukemic cells remain dependent on cytokines for growth [42]. In order to establish long-term in vitro AML cocultures, the addition of exogenous human cytokines was required [43], [44], and also in vivo engraftment of human primary AML samples in NOD-SCID mice has been shown to be facilitated by the addition of human cytokines [45], [46]. Yet, the specific cytokine-dependency of individual subtypes of AML has not been studied in detail, and cytokine-induced signal transduction in leukemic stem cell-enriched populations versus more committed leukemic progeny is only beginning to be examined. We observed that cytokine-induced signal transduction is highly variable between primary AML samples, differing significantly from normal hematopoietic cells. TPO, G-CSF, IL-3 and GM-CSF were the most dominant cytokines that induced STAT5 phosphorylation, although clear specificity existed in individual responses as well. When CD34^+^/CD38^−^ HSCs, CD34^+^/CD38^+^ MPPs and CD34^−^ subfractions were taken into account separately, we found differential responses throughout samples as well. For example, AML 2005-289, 2003-152, 2005-258 and 2007-046 responded to cytokines especially in CD34^+^ fraction (HSCs and MPPs) but not in the CD34^−^ fraction. Meanwhile, in other AML cases it was particularly the CD34^−^ population that responded to cytokine stimulation (e.g. AML 2003-119 or 2003-114). In the majority of the cases, comparable responses were detected between HSCs and MPPs. It needs to be pointed out that even though we followed the same definitions for HSC, CMP, GMP and MEP in AML samples as in healthy CB and PB using CD34, CD38, CD123 and CD45RA as antigens, these definitions of stem and progenitor compartments might not be valid in AML due to alterations in antigen expression. These responses could not be predicted by the presence of receptors, since in some cases no signaling could be observed in response to a certain cytokine despite expression of its receptor, while in other cases relatively low levels of receptor expression correlated with a strong cytokine-induced STAT5 response. Also, in 4 out of 5 cases, long-term expansion on MS5 bone marrow stroma could only be established in the presence of exogenous cytokines. In some cases, the addition of only IL-3 (AML 2006-014) or G-CSF (AML 2003-119) was sufficient to drive long-term expansion, but a clear (additive or in some cases synergistic) cooperation was observed between TPO, IL-3 and G-GSF. Clearly, the strong cytokine-induced STAT5 phosphorylation that was observed by e.g. TPO in AML 2006-014 or IL-3 in AML 2005-289, was not sufficient to drive long-term expansion. Apparently, besides phosphorylation of STAT5, activation of additional signal transduction pathways was required.

In 5 out of 10 cases, we observed constitutive phosphorylation of STAT5. Three of those cases expressed the FLT3 internal tandem duplication (FLT3-ITD), but in two other FLT3-ITD cases no constitutive STAT5 phosphorylation could be observed. Apparently, not in all FLT3-ITD cases STAT5 is constitutively activated, while in some FLT3-wt AMLs constitutive STAT5 phosphorylation may be observed as consequence of the autocrine production of growth factors [20], [47]. We did observe strongly reduced cytokine responses in those AMLs characterized by constitutive STAT5 activation. The cytokine-induced signal transduction pathways are tightly regulated by negative feedback regulators, including protein tyrosine phosphatases such as SHP-1, the protein inhibitors of activated STATs (PIAS) and the suppressor of cytokine signaling (SOCS) proteins [48]. The SOCS family (SOCS1-7 and CIS) negatively modulates the JAK-STAT pathway by inactivation of JAKs, blocks access of STATs to receptor binding sites and targets proteins for ubiquitination and degradation. Our gene expression profiling revealed that SOCS2 level was significantly higher in AMLs with constitutive STAT5 activation than those without (data not shown), suggesting a negative feedback loop was indeed involved in case of constitutively activated STAT5, rendering those cells less sensitive to further cytokine stimulation.

In conclusion, we find that intracellular FACS analysis is an informative tool to study cytokine-induced STAT5 phosphorylation at a single-cell level, in both normal and leukemic stem/progenitor cells. This platform will allow a further detailed analysis of the mechanisms by which cytokine signaling contributes to the process of leukemic transformation in specific leukemic stem and progenitor cell subpopulations.

## Materials and Methods

### Reagents and Antibodies

Paraformaldehyde (2%) (Sigma, Zwijndrecht, The Netherlands) was dissolved in H_2_O and 10% PBS and filtered over 0.45 µm filters (Millipore, Etten Leur, The Netherlands). Methanol (Merck, Schiphol-Rijk, The Netherlands) was diluted to 90% (v/v) in PBS. BSA was dissolved in PBS at 10% and filtered over 0.45 µm filters. All the above reagents were aliquoted and stored at −20°C. The following antibodies were used for FACS analyses: Alexa Fluor 647 mouse anti-Stat5 (pY694) (BD Biosciences, Breda, The Netherlands, cat. 612599, 20 µl per sample), CD34-FITC (BD Biosciences, Cat. 555821, 10 µl per sample), CD38-PerCP/Cy5.5 (BioLegend, Uithoorn, The Netherlands, Cat. 303522, 3 µl per sample), CD123-PE (BioLegend, Cat. 306006, 10 µl per sample), CD123-PE-Cy7 (BioLegend, Cat.306010, 1 µl per sample), CD45RA-PE-Cy7 (BD Biosciences, Cat. 337186, 0.1 and 1 µl for per fixed and unfixed sample), CD45RA-pacifc blue (BioLegend, Cat.304118, 5 µl per sample), CD110-APC (BD Biosciences, Cat. 551314, 7 µl per sample), CD114-PE (BD Biosciences, Cat. 554538, 5 µl per sample), CD116-PE (BD Biosciences, Cat. 551373, 5 µl per sample), mouse IgG1 κ isotype control labeled with APC (BD Biosciences, Cat. 555751, 7 µl per sample), PE (BioLegend, Cat.400114, 5 µl per sample) and Alexa 647 (BD Biosciences, Cat. 557732, 20 µl per sample). Antibodies used for Western blot were phospho-STAT5 (Tyr694) (BD Biosciences, Cat. 611964), phospho-p44/42 MAPK (Thr202/Tyr204) antibody (Cell Signaling, Leiden, The Netherlands, Cat. 9106), phospho-p70 S6 Kinase (Thr389) antibody (Cell Signaling, Cat. 9205), phospho-STAT3 (Tyr705) (Cell Signaling, Cat. 9131) and ERK1 antibody (Santa Cruz Biotechnology, CA, USA, Cat. SC-94). Antibodies against the FcR were used to prevent aspecific binding. (Miltenyi Biotec, Utrecht, The Netherlands)

### Isolation of Normal and Leukemic CD34^+^ Cells

Normal CD34^+^ cells were derived from neonatal cord blood (CB) from healthy full-term pregnancies from the Obstetrics departments of the Martini Hospital and University Medical Center Groningen after informed verbal consent. AML PB blasts from untreated patients were studied after informed verbal consent. All protocols were approved by the Medical Ethical Committee of the UMCG, Groningen, The Netherlands. Our Medical Ethical Committee decided that an informed verbal consent is sufficient in order to perform studies with CB and AML samples. After Ficoll separation of mononuclear cells (MNCs), CB CD34^+^ cells were enriched by magnetically activated cell sorting CD34 progenitor kit (Miltenyi Biotec). Alternatively, AML MNCs were stored in liquid nitrogen and AML CD34^+^ cells were isolated by MoFlo sorting (Dako Cytomation, Carpinteria, CA, USA).

### Cytokine Stimulation and Intracellular STAT5 Phosphorylation Analysis with FACS

CB CD34^+^ cells were grown in HPGM (Cambrex, Walkersville, MD) supplemented with 100 ng/ml Stem Cell Factor (SCF; Amgen, USA), FLT3 Ligand (FL; Amgen, USA) and Thrombopoietin (TPO; Kirin, Tokyo, Japan) at 5×10^5^ cells/ml at 37°C and 5% CO_2_. After 3 days, cells were washed twice with PBS and cytokine-depleted in HPGM at 8×10^5^ cells/ml overnight at 37°C. Primary AML MNCs and PB CD34^+^ cells were suspended in HPGM at 1.5×10^6^ cells/ml for 2 hours at 37°C. Cells were stimulated with cytokines for 15 minutes at 37°C after which fixation was performed with 2% PFA at a 1∶1 dilution directly into the medium at room temperature for 10 minutes. Cells were washed with 2% BSA/PBS twice and permeabilized with 1 ml 90% methanol on ice for 30 minutes. The permeabilized cells were washed twice, followed by FcR blocking (1 µl per 5×10^5^ cells) at 4°C for 10 minutes. Samples were stained in 100 µl final reaction volumes with the following antibodies at room temperature for 1 h: phospho-STAT5-Alexa 647; CD34-FITC; CD38-Percp/Cy5.5; CD123-PE; CD45RA-PE-Cy7. Samples were washed twice after incubation and analyzed on an LSR II flow cytometer (BD). Data were analyzed using WinList 6.0 (Topsham, ME, USA) and FlowJo (TreeStar, OR, USA) software.

The K562 cell line was cultured in RPMI 1640 medium (Biowhittaker, Verviers, Belgium) supplemented with L-glutamate, 10% fetal calf serum (FCS; Sigma) and 1% penicillin/streptomycin (P/S). The UT-7 cell line was cultured in IMD medium (PAA Laboratories GmbH, Pasching, Austria) with 10% FCS and 1% P/S, supplemented with 10 ng/ml granulocyte macrophage colony-stimulating factor (GM-CSF; Genetics Institute, Cambrigde). The UT-7 cells were stimulated with 10 U/ml Erythropoietin (EPO; Cilag; Belgium) as indicated in the text. Cells were used for STAT5 phosphorylation by FACS as described above.

### Long-Term Cultures on MS5 Stromal Cells

4×10^4^ AML CD34^+^ cells were plated in 12-well plates precoated with MS5 stromal cells as described previously [43], [44]. Briefly, cells were expanded in LTC medium including αMEM (Fisher Scientific Europe, Emergo, The Netherlands) supplemented with heat-inactivated 12.5% FCS, heat-inactivated 12.5% horse serum (Sigma), 1% P/S, 2 mM glutamine, 57.2 µM β-mercaptoethanol (Sigma) and 1 µM hydrocortisone (Sigma). The culture was supplemented with either no cytokines or 20 ng/ml IL-3, granulocyte colony-stimulating factor (G-CSF; Rhone-Poulenc Rorer, Amstelveen, The Netherlands), TPO or in combination as described in the text. Cultures were kept at 37°C and 5% CO_2_ and demi-depopulated weekly for analysis.

### Cell Lysis and Western Blotting

Whole cell lysates were prepared of 3×10^5^ cells by resuspending and boiling in sample buffer (containing 2% SDS, 10% glycerol, 2% β-mercaptoethanol, 60 mM Tris-HCL pH 6.8 and bromophenol blue) for 5 minutes. Protein aliquots were separated by 10% SDS-PAGE and transferred to nitrocellulose membranes (Millipore) using a semidry electroblotter from Biorad (Veenendaal, The Netherlands). The membranes were blocked in PBST containing 5% nonfat milk prior to incubation with primary antibodies. Detection was performed with horseradish peroxidase-conjugated secondary antibody (Dako Cytomation, Glostrup, Denmark) and enhanced chemiluminescence (ECL) reagent (Roche Diagnostics, Basel, Switzerland).

## Supporting Information

Figure S1 Intracellular FACS analysis to study cytokine-induced (EPO) and oncogene-induced (BCR-ABL) STAT5 tyrosine phosphorylation in human hematopoietic cells. (A–C) The UT-7 cell line was cytokine-depleted overnight from GM-CSF and subsequently stimulated with EPO (10 U/ml). (A) The cells were stimulated for the indicated time points, harvested and cell extracts were Western blotted using antibodies against phospho-STAT5 (Y694) and total STAT5 protein. (B) The cells were stimulated for the indicated time points, fixed with paraformaldehyde (PFA) and permeablized with ice-cold 90% methanol, followed by staining with Alexa 647 labeled antibodies against phospho-STAT5 (Y694). The histograms representing activated STAT5 are shown. (C) Graphic representation of the mean fluorescence intensity (MFI) of the experiment shown in B (solid line, with fill) and quantified Western blotting results from A (dotted line, no fill). (D–F) The BCR-ABL positive K562 cell line was cultured in RPMI with 10% FCS and 1% P/S at 1x10E6/ml, treated with imatinib for 1 hour at increasing concentrations as indicated. Western blotting (D) and intracellular FACS (E) for STAT5 phosphorylation were performed. (F) Quantified Western blotting results from D (dotted line, no fill) and mean fluorescence intensity (MFI) values from E (solid line, with fill) are shown.
(0.49 MB TIF)

Figure S2 Titration of surface markers in both fixed and unfixed conditions. Antibodies against surface markers of CD34-FITC, CD34-PE, CD34-APC, CD38-Percp/Cy5.5, CD38-PE-Cy5, CD123-PE, CD123-PE-Cy7, CD45RA-PE-Cy7, CD45-Pacific blue and CD110-PE, CD110-APC were tested in CB CD34+ cells in both unfixed and paraformaldehyde/methanol (F/M)-treated (fixed) cells. FcR blocking was performed 10 minutes before staining at 4°C. The percentages of the positive cells at different concentrations of antibodies are shown.
(0.72 MB TIF)

Figure S3 Maintaining surface marker expression after fixation and permeabilization. (A) Antibodies against surface markers of CD34-FITC (10 µL), CD38-Percp/Cy5.5 (3 µL), CD123-PE (10 µL) and CD45RA-PE-Cy7 (1 µL for unfixed cells and 0.1 µL for fixed cells) were identified in CB CD34+ cells in both unfixed and F/M-treated cells, as tested in Supplemental Figure S2. The percentages of the positive cells are shown after optimal titration. (B) CB CD34+ cells were analyzed in both unfixed and F/M-treated conditions with all above antibodies. The gating procedure and the percentage of each cell population are shown.
(0.14 MB TIF)

Figure S4 STAT5 phosphorylation in AML cases. (A–J) Ten AMLs were analyzed for intracellular STAT5 phosphorylation. The mononuclear cells (MNCs) were thawed and suspended at 1.5x10E6/ml in HPGM for 2 hours at 37°C. Cells were stimulated with cytokines for 15 minutes, followed by intracellular FACS. (G–J) Data from 4 AMLs is shown (raw data and data presented as multiplied value of percentage and mean fluorescence intensity (MFI) from the cells with activated STAT5).
(1.58 MB TIF)

Figure S5 STAT5 phosphorylation in CD123low/neg HSCs (CD34+CD38-) and CD123high/pos HSCs. The HSC compartment was further gated by the expression level of CD123, within which STAT5 phosphorylation was analyzed as indicated.
(0.51 MB TIF)
