# Supplementary figures and images for: Single-Cell STAT5 Signal Transduction Profiling in Normal and Leukemic Stem and Progenitor Cell Populations Reveals Highly Distinct Cytokine Responses
Source: PLoS One. 2009 Nov 24;4(11):e7989. doi: 10.1371/journal.pone.0007989 (PMC2776352; doi:10.1371/journal.pone.0007989)

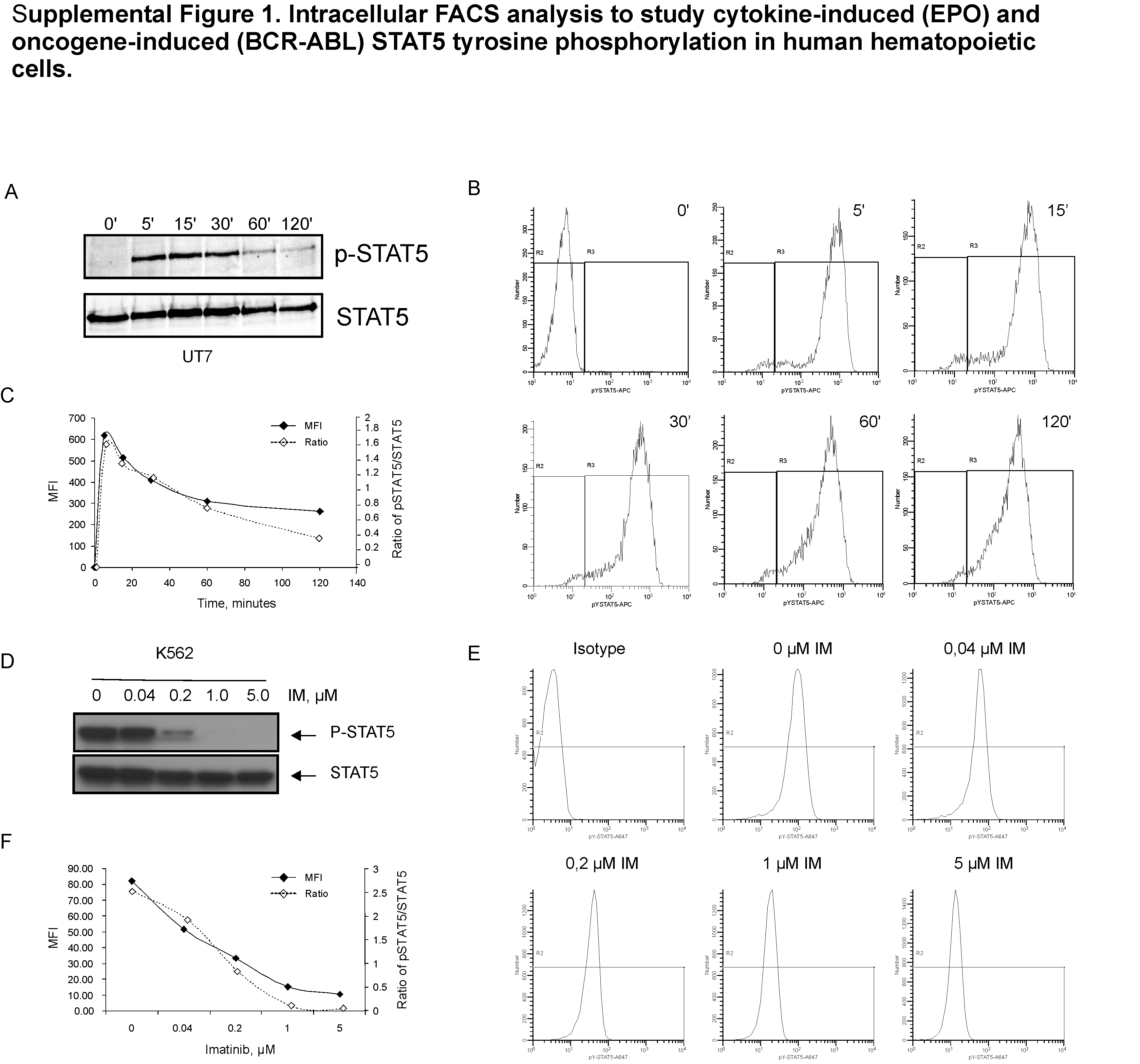

Supplement: Figure S1 — Intracellular FACS analysis to study cytokine-induced (EPO) and oncogene-induced (BCR-ABL) STAT5 tyrosine phosphorylation in human hematopoietic cells. (A–C) The UT-7 cell line was cytokine-depleted overnight from GM-CSF and subsequently stimulated with EPO (10 U/ml). (A) The cells were stimulated for the indicated time points, harvested and cell extracts were Western blotted using antibodies against phospho-STAT5 (Y694) and total STAT5 protein. (B) The cells were stimulated for the indicated time points, fixed with paraformaldehyde (PFA) and permeablized with ice-cold 90% methanol, followed by staining with Alexa 647 labeled antibodies against phospho-STAT5 (Y694). The histograms representing activated STAT5 are shown. (C) Graphic representation of the mean fluorescence intensity (MFI) of the experiment shown in B (solid line, with fill) and quantified Western blotting results from A (dotted line, no fill). (D–F) The BCR-ABL positive K562 cell line was cultured in RPMI with 10% FCS and 1% P/S at 1x10E6/ml, treated with imatinib for 1 hour at increasing concentrations as indicated. Western blotting (D) and intracellular FACS (E) for STAT5 phosphorylation were performed. (F) Quantified Western blotting results from D (dotted line, no fill) and mean fluorescence intensity (MFI) values from E (solid line, with fill) are shown. (0.49 MB TIF) [file pone.0007989.s001.tif]

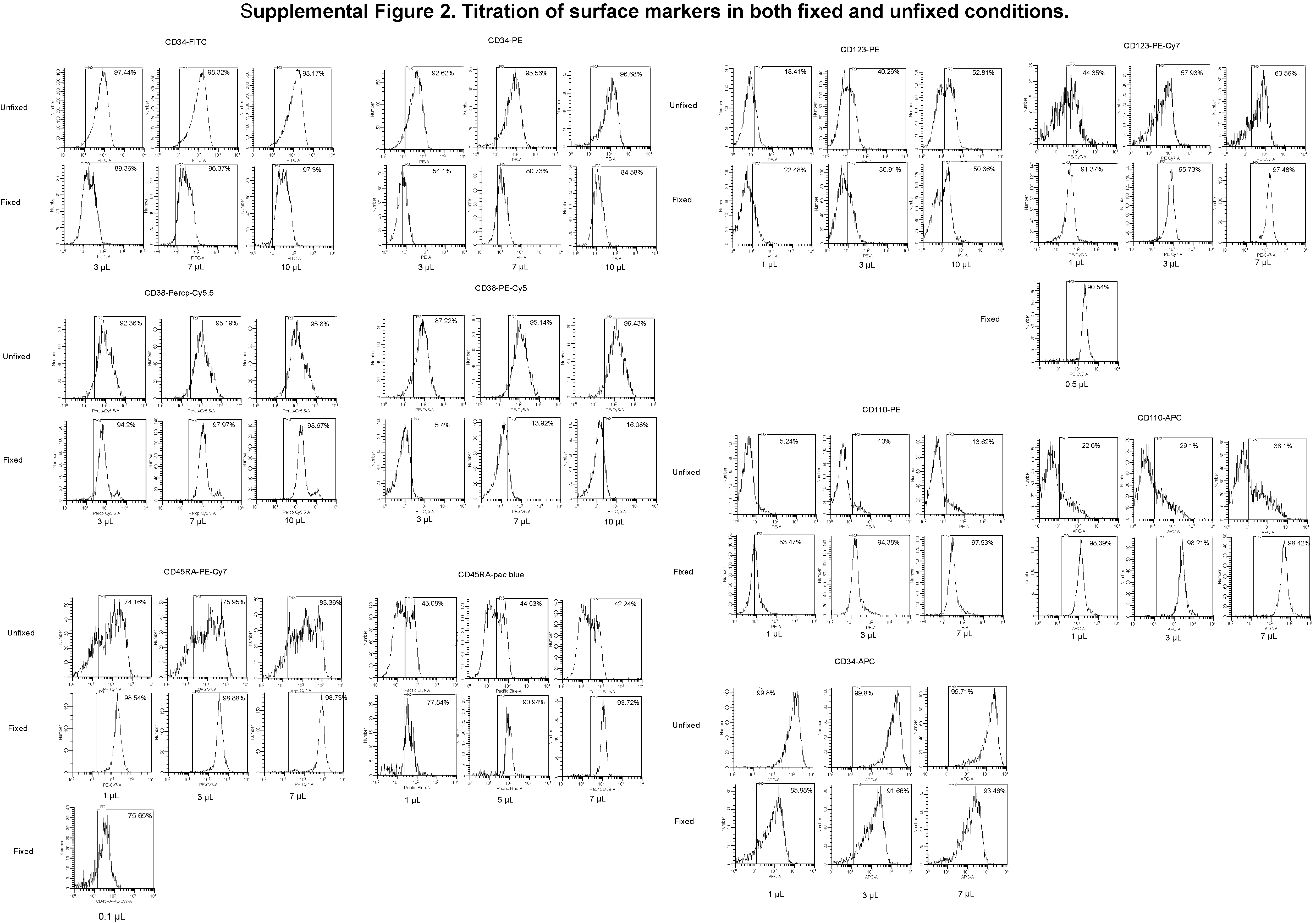

Supplement: Figure S2 — Titration of surface markers in both fixed and unfixed conditions. Antibodies against surface markers of CD34-FITC, CD34-PE, CD34-APC, CD38-Percp/Cy5.5, CD38-PE-Cy5, CD123-PE, CD123-PE-Cy7, CD45RA-PE-Cy7, CD45-Pacific blue and CD110-PE, CD110-APC were tested in CB CD34+ cells in both unfixed and paraformaldehyde/methanol (F/M)-treated (fixed) cells. FcR blocking was performed 10 minutes before staining at 4°C. The percentages of the positive cells at different concentrations of antibodies are shown. (0.72 MB TIF) [file pone.0007989.s002.tif]

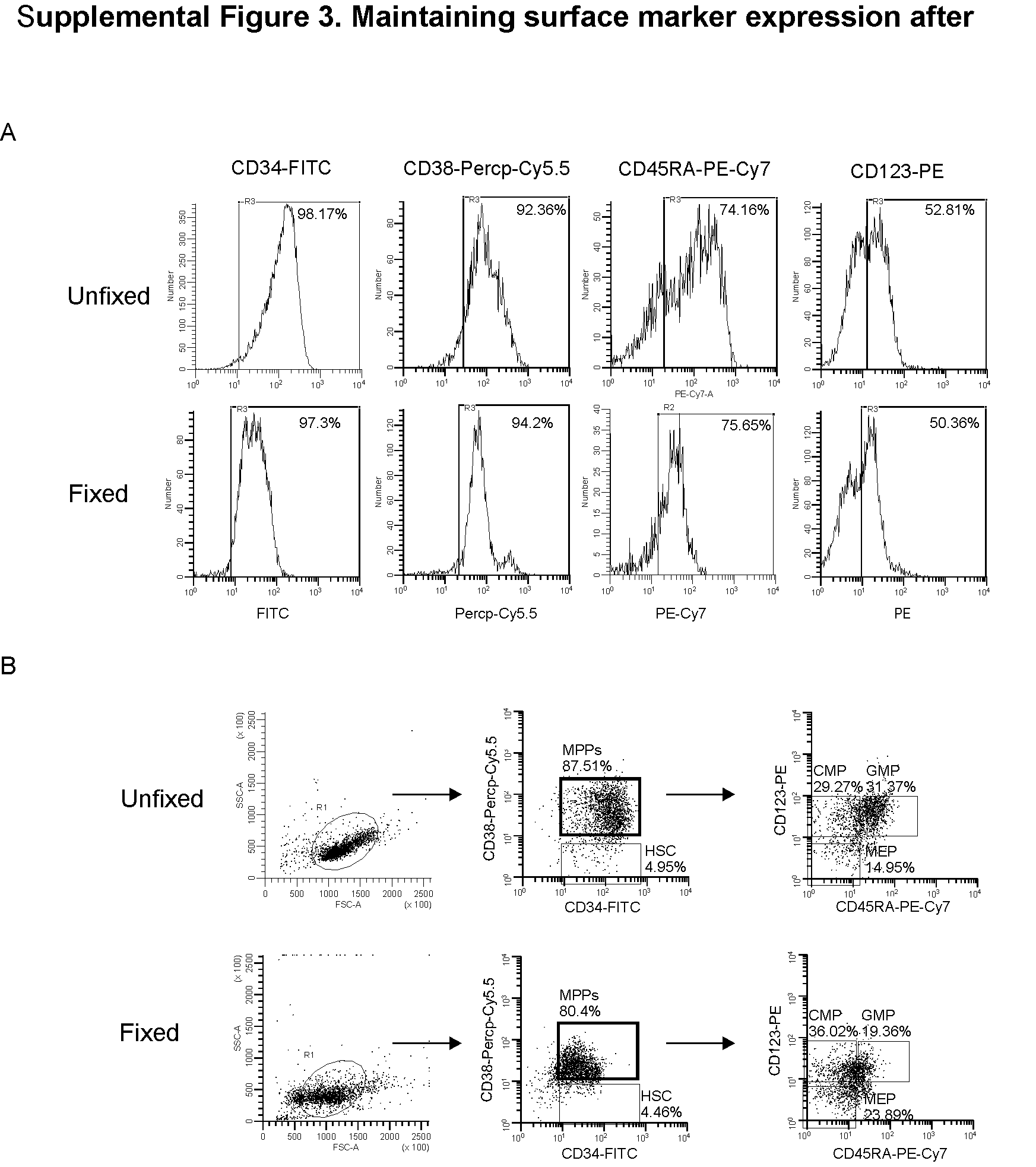

Supplement: Figure S3 — Maintaining surface marker expression after fixation and permeabilization. (A) Antibodies against surface markers of CD34-FITC (10 µL), CD38-Percp/Cy5.5 (3 µL), CD123-PE (10 µL) and CD45RA-PE-Cy7 (1 µL for unfixed cells and 0.1 µL for fixed cells) were identified in CB CD34+ cells in both unfixed and F/M-treated cells, as tested in Supplemental Figure S2. The percentages of the positive cells are shown after optimal titration. (B) CB CD34+ cells were analyzed in both unfixed and F/M-treated conditions with all above antibodies. The gating procedure and the percentage of each cell population are shown. (0.14 MB TIF) [file pone.0007989.s003.tif]

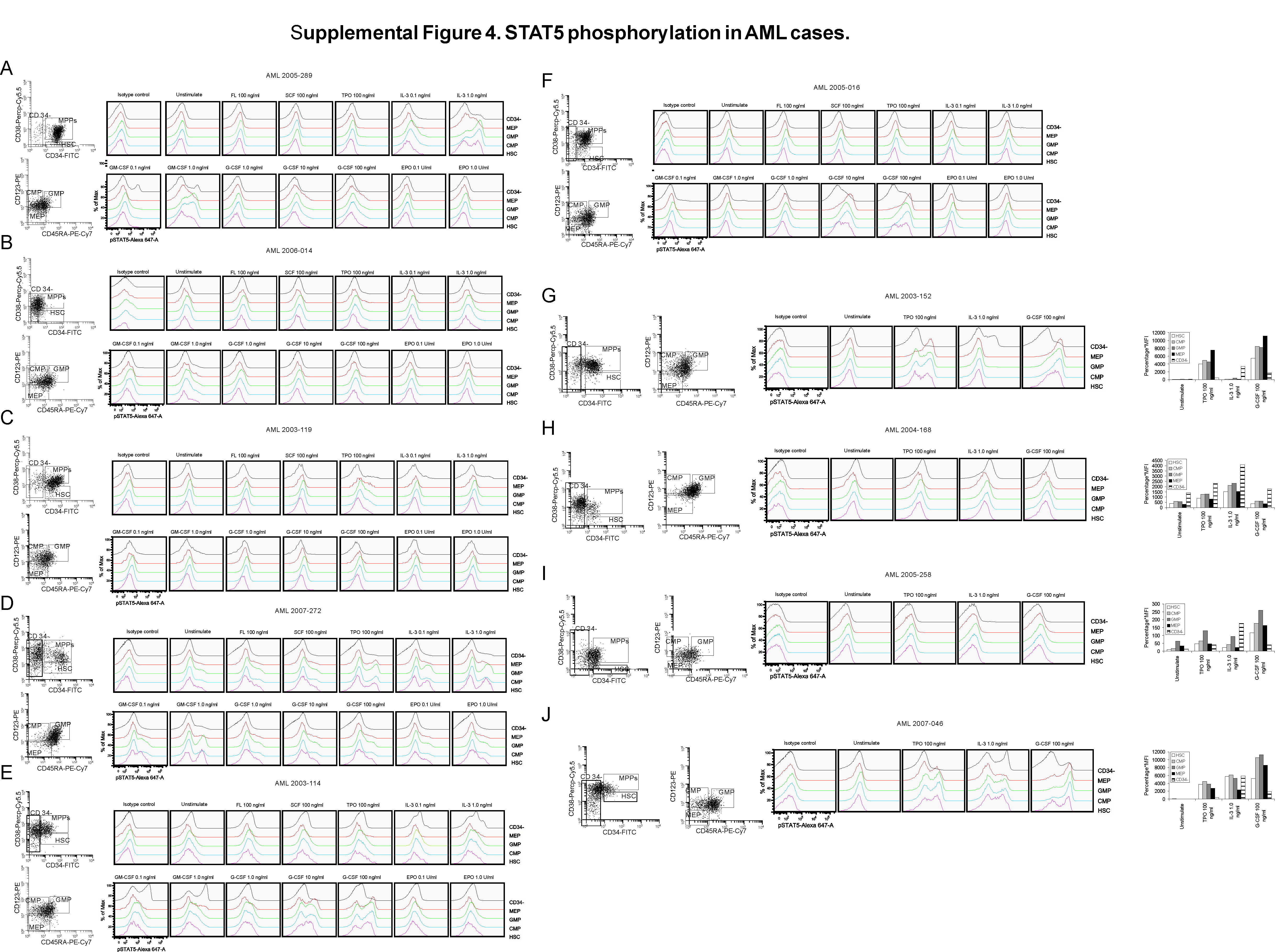

Supplement: Figure S4 — STAT5 phosphorylation in AML cases. (A–J) Ten AMLs were analyzed for intracellular STAT5 phosphorylation. The mononuclear cells (MNCs) were thawed and suspended at 1.5x10E6/ml in HPGM for 2 hours at 37°C. Cells were stimulated with cytokines for 15 minutes, followed by intracellular FACS. (G–J) Data from 4 AMLs is shown (raw data and data presented as multiplied value of percentage and mean fluorescence intensity (MFI) from the cells with activated STAT5). (1.58 MB TIF) [file pone.0007989.s004.tif]

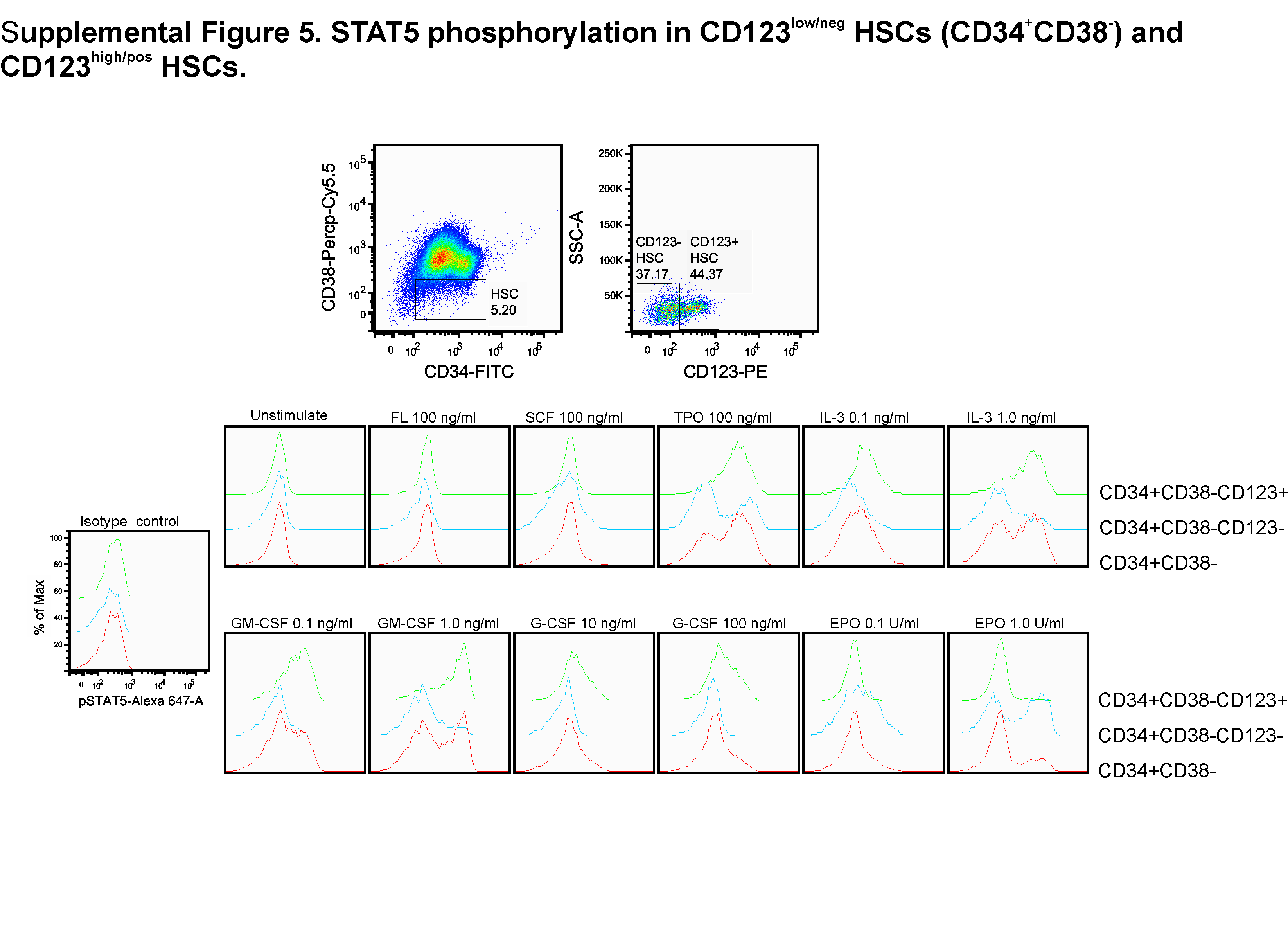

Supplement: Figure S5 — STAT5 phosphorylation in CD123low/neg HSCs (CD34+CD38-) and CD123high/pos HSCs. The HSC compartment was further gated by the expression level of CD123, within which STAT5 phosphorylation was analyzed as indicated. (0.51 MB TIF) [file pone.0007989.s005.tif]
